# Ultrasound-guided nerve hydrodissection of cervical nerve roots for cervical radicular pain in patients with mild and moderate to severe stenosis: a retrospective cohort study

**DOI:** 10.1038/s41598-023-40376-2

**Published:** 2023-08-24

**Authors:** Chang-Hao Lin, Yun-Shan Yen, Cheng-Yi Wu

**Affiliations:** 1 Department of Orthopaedics, Ditmanson Medical Foundation Chia-Yi Christian Hospital, Chiayi, Taiwan; 2 Department of Rehabilitation Medicine, Ditmanson Medical Foundation Chia-Yi Christian Hospital, Chiayi, Taiwan; 3 Department of Nursing, Chung Jen Junior College of Nursing, Health Science and Management, Chiayi, Taiwan

**Keywords:** Medical research, Outcomes research

## Abstract

Because fascial entrapment neuropathy can occur in multiple locations, ultrasound-guided nerve hydrodissection has become a key component of the treatment of cervical radicular pain. In this paper, we propose a combination of injectates used for nerve hydrodissection of the cervical nerve roots and compare the clinical outcomes of this treatment among patients with different severities of stenosis. This is a retrospective cohort study designed to compare outcomes between patients with mild stenosis and moderate to severe stenosis. Forty-four patients with mild cervical stenosis and 30 patients with moderate to severe cervical stenosis were consecutively enrolled into two groups. A 10-mL mixture in a single level consisting of 5% in Dextrose, 0.2% lidocaine (Xylocaine), and 4 mg betamethasone (Rinderon) was used for nerve roots hydrodissection. The two groups were compared with regard to their numeric rating scales (NRS) of pain, proportion of patients who exhibited a favorable outcome (a reduction of pain ≥ 50%), duration of patient exhibited a favorable outcome, and occurrence of serious complications and minor side effects. The follow-up period ranged from 3 to 20 months. The NRS of both groups improved significantly by 1 week, 1 month, 3 months, and final follow-up after the initial injection. Differences in the groups’ NRS, proportion of patients who exhibited a favorable outcome, duration of patient exhibited a favorable outcome**,** and occurrence of serious complications and minor side effects were nonsignificant. There were 4 patients (5.4%) experienced dizziness in that resolved without further treatment. Ultrasound-guided nerve hydrodissection of cervical nerve roots is a safe procedure that reduces pain associated with cervical radicular pain, even in patients with moderate to severe stenosis.

## Introduction

Ultrasound-guided selective nerve root blocks have been used for treating patients with cervical radicular pain^1–6^. However, because fascial entrapment neuropathy can occur in multiple locations^7^, the effect of a block in a specific location may only partially relieve a patient’s pain. Therefore, separating the compressed nerve from the surrounding fascia and soft tissues to prevent adhesion or obstruction is crucial. Nerve hydrodissection involves injecting a large volume of solution into areas of suspected compression to ultimately release the compressed nerve. Several injectates, including normal saline, local anesthetics, corticosteroids, 5% dextrose in water, hyaluronidase, and platelet-rich plasma, have been used for hydrodissection, and the pharmacological effects of some of these injectates result in pain alleviation^8–10^. Because nerve hydrodissection reduces mechanical compressions in the target area, its effects may persist for extended periods.

Randomized controlled trials have reported the effectiveness and safety of nerve hydrodissection in carpal tunnel syndrome^11–19^ and ulnar neuropathy at the elbow^20^. One study with a small sample size described reproducible procedures for hydrodissection of the cervical spine nerve root^21^. The effectiveness and safety of cervical nerve root hydrodissection has yet to be thoroughly investigated.

Given that the benefit of nerve hydrodissection lies in reducing mechanical compression, the severity of the mechanical compression of the cervical nerve roots may affect a patient’s response to nerve hydrodissection. We hypothesized that patients with severe stenosis of the cervical nerve roots would experience less favorable clinical outcomes than those with only mild stenosis.

In this study, we present our combination of injectates used for ultrasound-guided nerve hydrodissection of cervical nerve roots, and compare the clinical outcomes of this treatment among patients with different severities of stenosis with a minimum follow-up period of 3 months.

## Materials and methods

In this retrospective cohort study, we consecutively enrolled patients from March 2017 to June 2021 in a single hospital. This study was performed in accordance with the guidelines of the Declaration of Helsinki. The study design was approved by Ditmanson Medical Foundation Chia-Yi Christian Hospital institutional review board (approval no.: 2021111), and the institutional review board waived the requirement to obtain the informed consent. The inclusion criteria comprised cervical radicular pain that had lasted for ≥ 3 months and limited pain relief after 1-month physical therapy and analgesic drugs. The exclusion criteria were as follows: prior cervical injections within 6 months, a history of cervical surgery, cervical malformation, systemic lupus erythematosus, Sjögren's Syndrome, rheumatoid disorders, and a follow-up period < 3 months. A total of 74 patients were enrolled and divided into 2 groups according to the severity of their stenosis as determined using T2-weighted magnetic resonance imaging and Kang’s grading system^22^ for cervical canal stenosis: group I (mild stenosis), which comprised 44 patients with mild stenosis (Kang grade 1), and group II, which comprised 30 patients with moderate to severe stenosis (Kang grade 2–3).

The injection method is described briefly as follows. The procedures evaluated in this study were performed by a single physician with more than 6 years of experience of performing ultrasound-guided procedures. All the treatments were performed as outpatient procedures. For the ultrasound-guided nerve hydrodissection, a GE Logiq S8 Ultrasound System® (GE Healthcare, Milwaukee, Wisconsin, USA) with a 3–12-MHz linear probe was used. With the patient in a supine position and their head externally rotated 30–40° from the targeted area, the patient’s frontal cervical spine area from the clavicle to the mandible was adequately disinfected with povidone-iodine (Betadine)^23,24^. Subsequently, the skin was allowed to dry for 30 s before preparing the aseptic draping for the procedure. The probe was covered by the sterile polyurethane film, and the inner surface was filled with ultrasound transmission gel. The 2% Chlorhexidine Gluconate (w/v) in 75% alcohol (v/v) was used for second disinfection of the injection site and the contact media for the ultrasound-guided hydrodissection. An in-plane approach was adopted, with the needle advancing from the posterior to anterior and lateral to medial directions.

**Figure 1:**
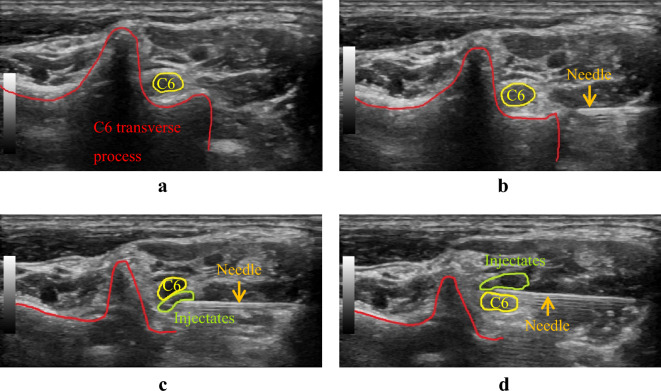
Procedures for C6 nerve root hydrodissection. (**a**) C6 transverse processes with anterior and posterior tubercles. (**b**) Hydrodissection while needle approaching the C6 nerve root. (**c**) Place the tip of the needle on the dorsal side of the C6 nerve root and inject a mixture of injectates. (**d**) Place the tip of the needle on the ventral side of the C6 nerve root and inject a mixture of injectates.

The hyperechoic anterior and posterior tubercles of the cervical vertebra served as bony landmarks in this study. The C5–C8 nerve roots were identified using the ultrasound by the shape of the transverse processes; the C5 and C6 transverse processes both have obvious anterior and posterior tubercles, C7 has no anterior tubercle, and C8 has no anterior nor posterior tubercle. The targeted transverse process was identified by slowly moving the probe in all directions using the C7 transverse process as a reference point. Optimal images of the nerve root, radicular artery, and surrounding vessels near the border of the nerve root were obtained through probe manipulation and power-mode color Doppler ultrasonography. Subsequently, a 50-mm 23G needle was inserted from the posterior and directed anteriorly toward the nerve root in the short-axis view by using the in-plane approach with the free hand technique. The physician placed the tip of the needle on the dorsal side of the nerve to hydrodissect the soft tissue around cervical nerve roots to the point where the injectate surrounds the entire nerve root while using simulating doppler color flow images to avoid possible damage to the spinal branch of deep cervical artery and the vertebral artery near the hydrodissection area and locating the area away from the radicular artery. After confirming the absence of abnormal findings and performing careful aspiration, the physician injected a 10-mL mixture in a single level of 5% in Dextrose, 0.2% lidocaine (Xylocaine), and 4 mg of betamethasone (Rinderon) under real-time ultrasound guidance to hydrodissect the nerve root to reduce the patient’s pain with fluid surrounding the nerve root. Figure 1 shows the ultrasound view of the C6 nerve root hydrodissection.

After the injection, the patient was monitored for the onset of clinical manifestations, such as mid-neck and contralateral arm pain, a metallic taste, dizziness, tachycardia, full-body paresthesia, auditory changes, slurred speech, or motor ataxia, for 10 min. Patients were scheduled to receive 3 consecutive injections at 3-week intervals. No other physical therapies or analgesic drugs were administered after the initial injection.

The primary outcomes were the reduction of pain in the neck, shoulders, and upper limbs. The numeric rating scale (NRS, 0–10) was used to quantify the pain reduction, and the proportions of patients who exhibited a favorable outcome (an improvement of pain ≥ 50%) were calculated at 4 time points (the 1-week, 1-month 1, 3-month, and final follow-ups). To assess the persistence of the treatment effect, the duration of each patient’s exhibition of a favorable outcome was recorded. Although the follow-up periods of all of the patients were ≥ 3 months, these periods were not equal. Therefore, the counting of days undergoing favorable outcomes might be limited. To account for this variation in follow-up duration, we calculated the persistence of favorable outcome using the days of exhibition of a favorable outcome divided by the days of follow-up ($\frac{\mathrm{Days\,of\,exhibition\,of\,a\,favorable\,outcome }\,(\mathrm{days})}{\mathrm{Days\,of\,follow}-\mathrm{up }\,(\mathrm{days})}$). This calculation reflects how long patients were able to maintain the favorable outcome during the follow-up period. The secondary outcomes were serious complications (including vessel injection, dural puncture, and directed injury of the spinal cord) and minor side effects (including dizziness, headache, skin bruises, local tenderness, itchiness, and allergic reactions).

An independent *t* test and a Mann–Whitney *U* test were used to compare continuous variables between groups, and a chi-square test and Fisher’s exact test were used to compare discrete variables between groups. Repeated measures of NRS were compared among the different time points and between the groups by using the generalized estimating equation method. All analyses were performed using IBM SPSS 21.0 (IBM, Armonk, NY, USA).

## Results

**Figure 2:**
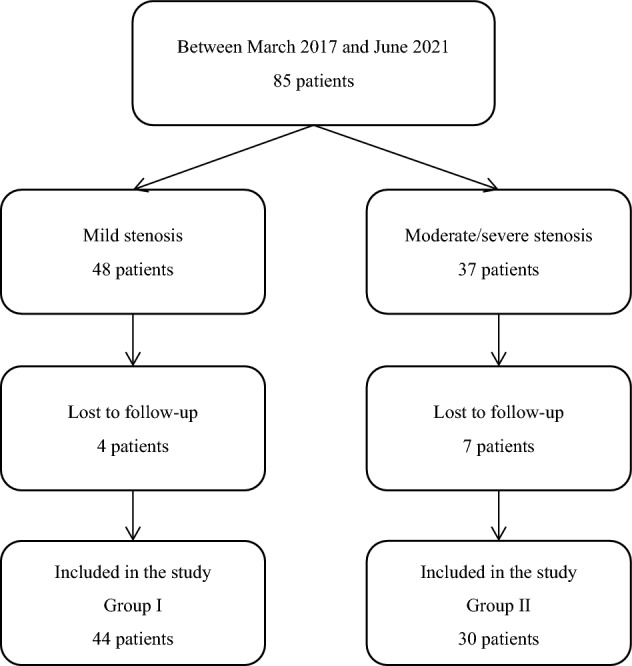
Flowchart of the selection process.

There were 85 patients met the inclusion criteria. Of these patients, 11 patients were excluded due to lost to follow-up (Fig. 2). Finally, a total of 74 patients were included in this study, of whom 44 (59.5%) were in group I (mild stenosis) and 30 (40.5%) were group II (moderate to severe stenosis). Table 1 presents the patient characteristics. Similar distributions of sex, age, pre-treatment pain score, and the number of injections were identified between the groups. The most commonly treated nerve root in both groups was C6 (90.9% of patients in group I and 96.7% of patients in group II), and most of patients underwent had multiple levels treated. The length of follow-up were 5.5 ± 3.8 (range, 3.0–20.0) months in group I, and 4.7 ± 3.1 (range, 3.0–15.0) months in group II.

**Table 1:** Patient characteristics.

	Group I mild stenosis	Group II moderate/severe stenosis	*p*-value
No. of patients	44	30	
Male	15(34.1%)	10(33.3%)	0.946
Age	57.0 ± 13.1	62.9 ± 12.0	0.052
Pain at pre-treatment (0–10)	6.4 ± 1.7	6.7 ± 2.0	0.556
No. of injections			
1	10(22.7%)	11(36.7%)	0.242^b^
2	12(27.3%)	3(10.0%)	
3	13(29.5%)	11(36.7%)	
4 and above	9(20.5%)	5(16.6%)	
Target nerve root			
Total no. of nerve roots	96	75	
C5, n (% of total no. of nerve roots)	32(33.3%)	18(24.0%)	
C6, n (% of total no. of nerve roots)	40(41.7%)	29(38.7%)	
C7, n (% of total no. of nerve roots)	22(22.9%)	21(28.0%)	
C8, n (% of total no. of nerve roots)	2(2.1%)	7(9.3%)	
Total no. of patients	44	30	
C5, n (% of total no. of patients)	32(72.7%)	18(60.0%)	
C6, n (% of total no. of patients)	40(90.9%)	29(96.7%)	
C7, n (% of total no. of patients)	22(50.0%)	21(70.0%)	
C8, n (% of total no. of patients)	2(4.5%)	7(23.3%)	
Multiple levels treated, n (% of total no. of patients)	37(84.1%)	25(83.3%)	1.000^b^
2 levels, n (% of total no. of patients)	23(52.3%)	10(33.3%)	0.110^b^
3 levels, n (% of total no. of patients)	13(29.5%)	10(33.3%)	
4 levels, n (% of total no. of patients)	1(2.3%)	5(16.7%)	
Length of follow-up (months)			
Mean ± sd	5.5 ± 3.8	4.7 ± 3.1	0.390^a^
Min − Max	3.0 − 20.0	3.0 − 15.0	

^a^Mann–Whitney *U* test.

^b^Fisher’s exact test.

**Figure 3:**
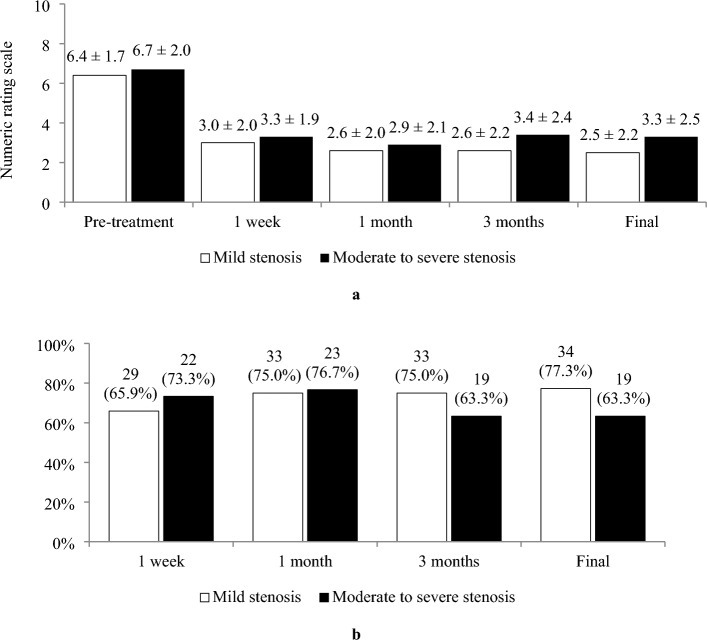
Pain reduction. (**a**) Numeric rating scale. (**b**) Proportions of patients who exhibited a favorable outcome (an improvement of pain ≥ 50%).

Before the injection, the mean NRS for pain were 6.4 ± 1.7 and 6.7 ± 2.0 in groups I and II, respectively. At 1 week after initial injection, the mean NRS had significantly decreased in both groups, and this effect persisted until the final follow-up (Fig. 3a). The improvements from pretreatment to 1 week, 1 month, 3 months, and final follow-up were not significantly different between the groups. During the follow-up period, of the 44 patients in group I, 29 (65.9%) had exhibited a favorable outcome by 1 week; the proportion increased to 75.0% at 1 month, remained the same until the 3-month follow-up, then increased to 77.3% at the final follow-up. For patients in group II, a similar trend was observed at 1 month, but an earlier decrease of proportion occurred by the 3-month follow-up. At the final follow-up, the proportion was 63.3%. No significant differences were identified between the groups at any of the time points (Fig. 3b).

By the final follow-up, 53 patients (71.6%) still exhibited a favorable outcome. After the initial injection, the mean persistence of favorable outcome were 78.6% ± 19.5% and 83.9% ± 19.6% in groups I and II, respectively; the groups did not exhibit a significant difference (Table 2). A total of 21 patients (28.4%) did not exhibit a favorable outcome by the final follow-up. There was no difference in the median persistence of favorable outcome between the two groups.

**Table 2:** Duration of patient exhibited a favorable outcome.

	Group I mild stenosis	Group II moderate/severe stenosis	*p*-value
Patients who exhibited a favorable outcome at the final follow-up			
No. of patients	34	19	
Persistence of favorable outcome			
Mean ± sd	78.6% ± 19.5%	83.9% ± 19.6%	0.270^a^
Median	83.8%	88.9%	
Patients who did not exhibit a favorable outcome at the final follow-up			
No. of patients	10	11	
Persistence of favorable outcome			
Mean ± sd	7.7% ± 10.2%	19.5% ± 24.8%	0.223^a^
Median	1.7%	5.4%	

^a^Mann–Whitney *U* test.

We did not observe serious complications in either group; however, 3 patients in group I and 1 patient in group II experienced transient dizziness that resolved without further treatment within 4 h. There was no association between the severity of stenosis and the incidence of complications.

Table 3 presents the risk factor analysis for unfavorable outcome at the final follow-up. In the favorable group, 9 (17.0%) of the patients did not receive repeated injections after the initial injection, and this percentage is significantly lower than the unfavorable group.

**Table 3:** Risk factor analysis for unfavorable outcome at the final follow-up.

	Favorable	Unfavorable	
	53	21	
Age	60.0 ± 12.2	58.6 ± 15.0	0.743
Pain at pretreatment (0–10)	6.5 ± 1.8	6.7 ± 1.9	0.758^a^
Stenosis			
Mild	34(64.2%)	10(47.6%)	0.192
Moderate/Severe	19(35.8%)	11(52.4%)	
Multiple levels treated	45(84.9%)	17(81.0%)	0.731^a^
No. of injections per year			
1	9(17.0%)	12(57.1%)	0.002^a^
2	10(18.9%)	5(23.9%)	
3	22(41.5%)	2(9.5%)	
4 and above	12(22.6%)	2(9.5%)	

^a^Fisher's exact test.

## Discussion

The patients who underwent ultrasound-guided nerve hydrodissection of the cervical nerve roots using our combination of injectates exhibited significant improvements in pain reduction, and the magnitudes of the improvements were similar in the patients with mild and moderate to severe stenosis. The persistence of the treatment effect and incidence of complications were not associated with the severity of stenosis.

Previous studies have reported significant alleviation of patients’ pain after ultrasound-guided selective nerve root blocks in the lower cervical spine. The mean NRS in six studies were 6.6, 2.3, 2.8, and 2.4 at the pretreatment, 1-month, 3-month, and 6-month follow-ups, respectively. The proportions of the patients reporting favorable outcomes (an improvement of pain ≥ 50%) were 80.9%, 71.1%, and 59.6% at the 1-month, 3-month, and 6-month follow-ups, respectively^2–6^. In our study, the overall mean NRS were 6.5, 2.8, 2.9, and 2.8 at the pretreatment, 1-month, 3-month, and final follow-ups, respectively. The overall proportions of patients who exhibited favorable outcomes were 75.7%, 70.3%, and 71.6% at the 1-month, 3-month, and final follow-up, respectively. Our results at the 1-month and 3-month follow-ups are compatible to those of previous studies. Among our patients, 20 were followed up for more than 6 months, and all of these patients achieved favorable outcomes at the final follow-up. Our treatment approach resulted in more favorable outcomes than did selective nerve root blocks at the 6-month follow-up. However, additional studies involving more cases are necessary to more thoroughly evaluate the long-term outcomes of the proposed treatment.

In our series, 68 (91.9%) of the patients reported alleviated pain immediately after the initial injection; 51 (68.9%) patients exhibited a favorable outcome within 1 week, and 56 (75.7%) reported a favorable outcome at 1 month, which indicates that the pain was gradually alleviated in some patients. The improvements in pain reduction, proportions of patients who achieved favorable outcomes, and persistence of pain reduction did not differ significantly between the patients with mild and moderate to severe stenosis at any of the time points. Therefore, the severity of a patient’s stenosis might not affect their response to nerve root hydrodissection.

In the present study, a mixture of 5% in Dextrose, 0.2% lidocaine (Xylocaine), and 4 mg of betamethasone (Rinderon) was used as the treatment injectate. The analgesic effect of 5% dextrose in water in the treatment of chronic neuropathic pain has been reported^21,25^, and related mechanisms might be able to reduce the activation of transient receptor potential vanilloid receptor-1 (TRPV1), which is associated with chronic neuropathic pain, and to decrease the discharge rate to reduce pain sensitivity and reverse energy-deficient states associated with neuropathy^25^. A low dose of lidocaine may be used to prevent patients’ discomfort during injection^21^. To facilitate the speed of onset of pain relief, the use of steroids may be considered; in this study, betamethasone (Rinderon) was selected because studies have reported its effectiveness and safety for use of cervical selective nerve root blocks^26,27^. A previous study has used a mixture of 5% dextrose in water and steroid for treatment of plantar fasciitis, and reported that 5% dextrose in water led to a significant reduction of pain during the intervention^28^. According to our results, the pain relief was adequate, and minor side effects seldom occurred, which verifies the rationale for using our combination^24,29,30^.

In this studies, a high percentage (83.8%, *n* = 62) of the patients underwent hydrodissection in multiple nerve roots simultaneously because they experienced pain in several locations that could not be attributed to a specific nerve root. Among the 12 patients who underwent hydrodissection in a single nerve root, 66.7% (*n* = 8) exhibited a favorable outcome by the final follow-up, and 1 patient (8.3%) experienced a minor side effect. Similar outcomes were found among the 62 patients who underwent multiple simultaneous nerve hydrodissection: 72.6% (*n* = 45) exhibited a favorable outcome at the final follow-up, and 3 (4.8%) experienced minor side effects. Although we hydrodissected multiple nerve roots of these patients simultaneously, the concentrations of medicines administered to each nerve root were low to prevent patients from experiencing side effects. Therefore, performing nerve hydrodissection in multiple nerve roots simultaneously could be considered as a treatment option when a patient’s symptoms involve multiple nerve roots.

In order to determine potential risk factors for unfavorable outcome, we compared patients who exhibit a favorable and unfavorable outcome at the final follow-up. Our result indicates repeated injections after the initial injection is the most important factor, and this finding is compatible to a previous study that reported that repeated injections performed at 2–3 weeks after the initial injection contributed to prolonged clinical benefits in patients of pain reduction^31^. Therefore, repeated injections may be recommended for patients.

This study has some limitations. The matched-group design was not employed, which decreased the comparability of the groups; however, we determined that analyzing the data of consecutive cases would allow the results to be more representative of the general population. Since a control group that underwent conservative treatment was not included in this study, the treatment effectiveness cannot be assessed. Considerable variation in the continuous data was observed, which indicates that the mean values for each variable may be inaccurate. Therefore, we investigated the persistence of favorable outcomes of the patients who achieved and did not achieve a favorable outcome by the final follow-up separately, and different distributions were identified between the 2 stratified groups. Further, a longer follow-up period will be needed to investigate the long-term results, and the findings should be verified through studies with larger sample size.

In conclusion, ultrasound-guided nerve hydrodissection of cervical nerve roots is a safe procedure that reduces pain associated with cervical radicular pain, and even patients with moderate to severe stenosis can achieve favorable outcomes from such treatment. Compared with the patients who underwent selective nerve blocks, the patients who underwent nerve hydrodissection exhibited similar outcomes at the 1-month and 3-month follow-ups. Our results indicates that a 10-mL mixture in a single level of 5% in Dextrose, 0.2% lidocaine (Xylocaine), and 4 mg of betamethasone (Rinderon) is appropriate for nerve hydrodissection of the cervical nerve roots. Hydrodissection of multiple cervical nerve roots can be performed when a patient’s symptoms are not attributable to a specific nerve root.

## Data Availability

The dataset used and/or analysed during the current study are available from the corresponding author on reasonable request.
